# Understanding the Renal Fibrotic Process in Leptospirosis

**DOI:** 10.3390/ijms221910779

**Published:** 2021-10-05

**Authors:** Luan Gavião Prado, Angela Silva Barbosa

**Affiliations:** 1 Laboratório de Bacteriologia, Instituto Butantan, Avenida Vital Brasil, 1500, São Paulo 05503-900, Brazil; luan.prado@esib.butantan.gov.br; 2 Departamento de Microbiologia, Instituto de Ciências Biomédicas, Universidade de São Paulo, Avenida Lineu Prestes 1374, São Paulo 05508-000, Brazil

**Keywords:** fibrosis, kidney fibrosis, CKD/CKDu, *Leptospira*, leptospirosis

## Abstract

Leptospirosis is a neglected infectious disease caused by pathogenic species of the genus *Leptospira*. The acute disease is well-described, and, although it resembles other tropical diseases, it can be diagnosed through the use of serological and molecular methods. While the chronic renal disease, carrier state, and kidney fibrosis due to *Leptospira* infection in humans have been the subject of discussion by researchers, the mechanisms involved in these processes are still overlooked, and relatively little is known about the establishment and maintenance of the chronic status underlying this infectious disease. In this review, we highlight recent findings regarding the cellular communication pathways involved in the renal fibrotic process, as well as the relationship between renal fibrosis due to leptospirosis and CKD/CKDu.

## 1. Introduction

Leptospirosis is an infectious and zoonotic disease caused by pathogenic bacteria of the genus *Leptospira*. These highly motile spirochetes, characterized by a helicoidal and thin shape, have two *endoflagella* that never cover the entire bacterial length, which is one of the most important and peculiar characteristics of these bacteria [1,2,3,4,5]. Worldwide, it has been estimated that 1.03 million cases and 58,000 deaths from leptospirosis are reported annually [6,7,8,9]. The disease has become an issue of concern in countries in Europe and other developed countries, either as an emerging or re-emerging condition. In developing countries, such as those in East Asia, South and Central America, and Sub-Saharan Africa, the disease has been considered to be neglected [6,10,11,12,13]. 

Natural disasters, rapid urbanization, and a lack of basic sanitization (e.g., water and sewage treatment or final garbage disposal) are considered risk factors for the occurrence of the disease, both in high- and low-income countries—but mostly the latter [8,14,15]. Professional occupation, gender, and age are also considered risk factors and are good predictors for leptospirosis. For example, living in a favela in Brazil or working in a rice plantation in an Asian country are high-risk factors for developing the disease and, as has been shown recently, chronic kidney disease [13,15,16]. 

Leptospirosis-related fibrosis, as a sequela from acute disease or due to persistent chronic infection and maintenance of bacteria in proximal convoluted renal tubules, remains overlooked and may be more associated to chronic kidney disease (CKD) than previously considered [13,16]. Kidney fibrosis is defined as accumulation of proteins from the extracellular matrix (ECM) in the interstitium and/or in the tubular basement membrane [17] and is the final outcome of many diseases (e.g., chronic kidney disease), characterized by the loss of architectural and functional roles. CKD (and, consequently, kidney fibrosis) affects around 10% of the global population. Unsurprisingly, countries with a high leptospirosis prevalence also have a high prevalence of CKD of unknown etiology [16,18,19].

A few pathways have been described as inducers or enhancers of kidney fibrosis, such as the transforming growth factor-β1 (TGF-β1) [20,21,22,23,24,25] and the Wnt (a portmanteau from Wingless and Int-1)/β-catenin signaling pathways [25,26,27,28,29,30], as well as the C3a/C3aR axis signaling pathway [31,32,33]. Additionally, imbalance and non-coordinated crosstalk between pathways are also related to kidney fibrosis. An example is illustrated by hepatocyte growth factor (HGF) and TGF-β1 imbalance, a well-described mechanism of induction of kidney fibrosis [34,35,36,37,38,39,40].

With that in mind, in this review, we describe the most important pathways involved in tissue fibrosis and summarize recent findings regarding leptospirosis-related renal fibrosis. Furthermore, we highlight the incidental concurrence of this infectious disease and the outbreak of CKDu globally. 

## 2. Kidney Fibrosis

As an overly complex process, kidney fibrosis in leptospirosis is approached in a stepwise manner for didactic purposes. First, we describe the pathways that are involved in fibrosis, the downstream effectors, and the outcomes of their activation, such as the epithelial–mesenchymal transition (EMT) and morphological and molecular patterns during this process and leptospiral infection. Then, we relate the pathways with the histological and gross alterations seen in kidney fibrosis during chronic leptospirosis. Finally, new approaches towards studying the relationship between leptospirosis and chronic kidney disease are discussed, as this subject has been a growing concern for nephrologists and *Leptospira* researchers. 

### 2.1. TGF-β1 Signaling Pathway

As reviewed elsewhere [41,42], TGF-β1 signaling has been exhaustively studied and implicated as one of the most important pathways involved in fibrosis in multiple organs, including the kidneys. This pathway is generically constituted by a TGF receptor ligand (cytokines, growth, and differentiation factors), type-II or -I TGF receptors, and downstream effector proteins (named Smads). TGF-β signal transduction plays a crucial role in both physiological and pathological conditions. 

Depending on the milieu and context of the cell, signaling by this pathway may have developmental effects during embryogenesis and promote tissue remodeling after injury but can also lead to fibrogenic effects when deregulated and overly activated [21,22,42,43,44,45]. Although it appears to be a simple pathway, due to the few actors, there exist a variety of mechanisms to control its activation, such as receptor ubiquitylation, sumoylation, and neddylation [46,47,48,49,50,51]; receptor glycosylation inhibition [52,53] and shedding [54]; and, finally, TGF-β latency mediated by the interaction between TGF-β, latency-associated polypeptides (LAP), latent TGF-β binding protein (LTBP), and the extracellular matrix [55,56,57]. 

In addition to post-translational modification inhibitory mechanisms, this pathway has inhibitory Smads that are activated downstream and interact with other Smads, abolishing or diminishing the response to certain stimuli [58,59,60]. Smad7 was first described in 1997 by Nakao et al. [61], as an intrinsic regulatory protein that inhibits the phosphorylation of mainly Smad2, but also Smad3, as well as preventing over-activation of the pathway. 

It also targets the TGFβ type-I receptor for ubiquitylation, leading to the receptor’s destruction by the proteasome, thus, decreasing the amount of receptors available in the plasma membrane and controlling the pathway’s activation [62]. Overexpression of Smad7 in rat tubular epithelial cells prevents Smad2 phosphorylation and the production of ECM proteins, such as collagen I, III, and IV [60]. Furthermore, Smad7-disrupted mice have more evident kidney fibrosis—induced by Unilateral Ureteral Obstruction (UUO)—compared to the WT group. Collagen I and III deposition and α-SMA production in Smad7-deficient mice confirm the role of Smad7 in controlling TGF-β1 activation and the development of fibrosis [63].

Other important TGF-β signaling pathway repressors are Sloan-Kettering Institute (Ski) and Ski novel (SnoN). Both of these have structural similarities, including a SAND domain (named after Sp100, AIRE-1, NucP41/75, and DEAF-1), which interacts with Smad4 and represses the pathway after its activation [64]. In a study using a mouse model and tubular epithelial cells to evaluate the participation of TGF-β1 in kidney fibrosis, the authors demonstrated that there was a reduction in SnoN and Ski during in vivo fibrosis and that downregulating them in tubular epithelial cells amplified the response to TGF-β1 activation. 

In line with a repressive role of these proteins on the TGF-β1 pathway, the ectopic expression of both in the cellular model rendered cells resistant to EMT and abolished the production of fibrosis markers, such as α-SMA [65]. Still, after stimulating HK-2 human renal proximal tubule cells with a hyperglycemic medium, the exogenous overexpression of SnoN and downregulation of Arkadia (a negative regulator of SnoN) can block EMT [66]. 

**Figure 1:**
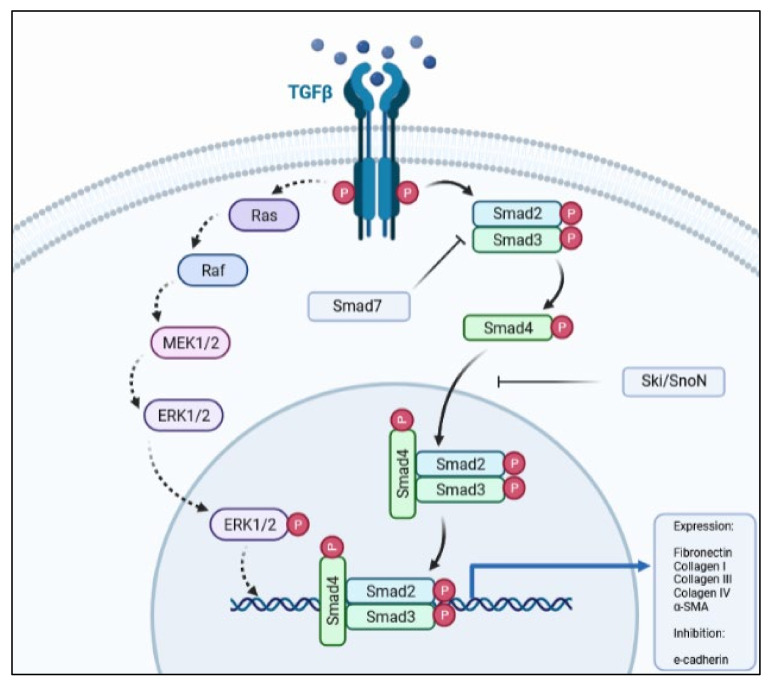
Simplified schematic representation of the TGF-β pathway, depicting the main proteins involved in signal transduction.

Although the pathway has its own regulators and repressors, there are other cytokines and growth factors that may enhance, be activated by (e.g., Wnt/β-catenin) [23,29], or antagonize (e.g., hepatocyte growth factor; HGF) the TGF-β1 signaling pathway. The crosstalk between the TGF-β1 and Wnt/β-catenin during EMT processes has been well-documented where the activation of these pathways leads to transition of the epithelial phenotype to a mesenchymal one. Other pathways, such as PI3K-Akt-mTOR and MAPK, and the phosphorylation of downstream proteins (e.g., MEK1/2 and ERK1/2) also respond to TGF-β1 stimulus (Figure 1), albeit to a lower degree than when activated by their own receptor’s ligands. Even so, these pathways play an important role in the occurrence of EMT, as their blockade abolishes or diminishes EMT in epithelial cells [42,67,68].

HGF is a pleiotropic growth factor mainly secreted by mesenchymal cells after an injury or an inflammatory process [37,69,70,71,72,73,74]. Its action in cells is mediated by the MET receptor, which triggers the downstream phosphorylation and activation of effector proteins (e.g., ERK), leading to the expression of target genes resulting in the production of metalloproteases 2 and 9 [75], SnoN, and Ski [64,65,66] and inducing myofibroblast apoptosis [75]. 

The exogenous administration of HGF in different animal models of fibrosis leads to less prominent or abolishes organ fibrosis in chronic kidney disease [34,35,36], unilateral ureteral obstruction [38,76], and diabetic nephropathy [40]. Beyond its evident role in antagonizing TGF-β1 and its antifibrotic activity, the anti-inflammatory potential of HGF may also contribute to the final effects in vivo and in vitro, as renal inflammation directly contributes to progression of chronic kidney disease [71,77,78,79,80]. This is further discussed in another section.

### 2.2. Wnt/β-Catenin Signaling Pathway

**Figure 2:**
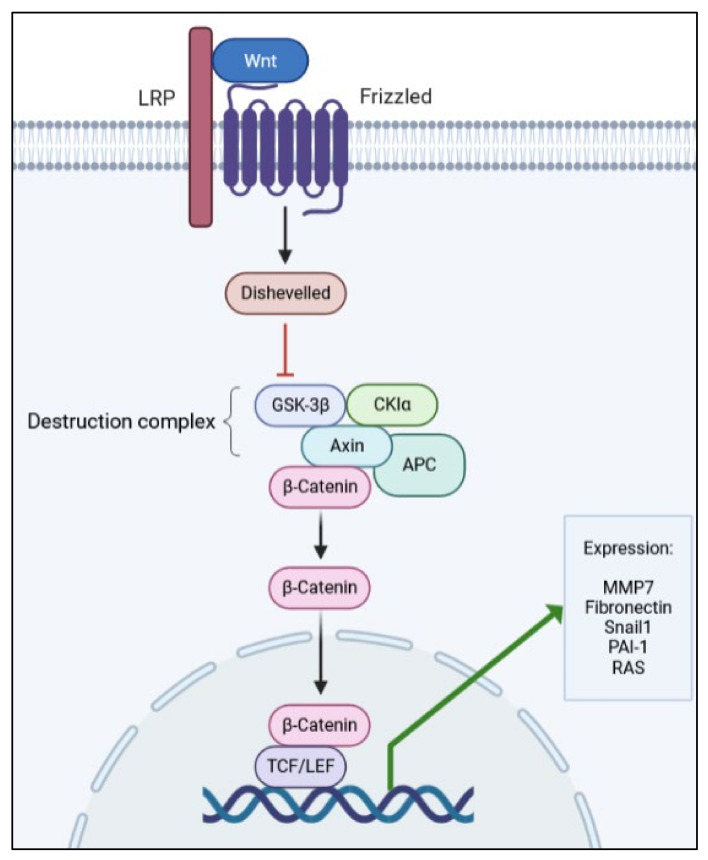
Simplified schematic representation of the Wnt/β-catenin pathway, depicting the main proteins involved in signal transduction.

Another apparently simple but complex pathway involved in renal fibrosis is the Wnt/β-catenin pathway. It is simple, in that it has a few actors from its activation until the outcome, which are a Wnt-protein ligand, a frizzled receptor (and its co-receptors), and dephosphorylated β-catenin (see Figure 2). On the other hand, it is complex as it may activate and be controlled by many other pathways, including signaling by TGF-β1 [27,28,30,81,82,83].

Until the past decade, studies on the Wnt signaling pathway focused mainly on cancer and its pharmacological treatment, and embryonic development. Colorectal cancer has been directly associated with mutations on important Wnt positive- and negative-regulators, and mutations on the main actors of the pathway cause abnormalities in embryo development, such as a lack of wings in *Drosophila melanogaster* and a lack of anterior cerebellum in mice [84]. Deregulation of this pathway has recently been associated with fibrosis, as a cause or consequence of tissue damage and pro-fibrotic stimulus [26,28,30,85,86,87].

In total, 19 Wnt ligands, which are glycoproteins that bind to the frizzled receptors to trigger activation, have been described [26,88]. The pathway comprises scaffold and adaptor proteins that mediate the interaction between the receptors and effector proteins. After Wnt ligand binding to the frizzled receptor, LDL-related receptor proteins 5 and 6 (LRP5/6) are phosphorylated and recruit Dishevelled proteins, which are responsible for inhibiting the destruction complex (Glycogen synthase kinase 3β, GSK-3β; Casein kinase Iα, CKIα; Axin; and Adenomatosis polyposis coli, APC). 

Then, stable dephosphorylated β-catenin is released from the complex, enters the nucleus, and interacts with LEF (lymphoid enhancer factor) and T-cell factor (TCF), leading to the expression of target genes of the pathway [84]. As with other signaling pathways, the Wnt pathway has its own regulators, thus, avoiding over-activation and pathological outcomes. One of the most important Wnt modulators is the Dickkopf family (DKK) of proteins [29,86,89], which binds to LRP5/6 co-receptors and prevents the binding of Wnt ligands to the Frizzled receptor, thus, blocking the pathway [90]. 

In a mouse model of kidney fibrosis caused by UUO, only three Wnt genes (Wnt5b, Wnt8b and Wnt9b) are not upregulated, with a similar expression in sham mice. The other 16 Wnt genes are upregulated, following different patterns throughout the course of the experiment. Some of them are overexpressed within the first week, reach a peak, and then expression levels decline over the next seven days; others are upregulated within the first week and expression levels are sustained throughout the 14-day experiment; and, in others, the expression levels increase progressively during the entire experiment. There is also an increase in cytoplasmic and nuclear β-catenin during the course of the assay, further reinforcing that the canonical pathway is truly activated, with a final response of ECM production and kidney fibrosis evident at day 14 after UUO [85]. 

Klotho is a membrane bound protein that has been implicated as a negative regulator of the Wnt/β-catenin pathway. In HK-2 cells, Klotho inhibition by si-RNA leads to a more evident EMT after TGF-β1 stimulation, showing that there is a crosstalk between both pathways. In an in vivo model of chronic allograft dysfunction, Klotho is downregulated by 24 weeks after transplantation [91]. Reduced Klotho expression was also reported in the folic acid model of fibrosis [92]. Non-canonical activation of this pathway occurs independently from Klotho activity since the MMP-7 activation of β-catenin is abrogated by an inhibitor of transcription of target genes, but not by Klotho [93]. 

### 2.3. C3a/C3aR Signaling Pathway

Complement system proteins play important roles in renal fibrosis. As part of the bloodstream’s innate immune system, the effectors and regulators of the classical, alternative, and lectin pathways of the complement system are mostly produced and secreted by the liver directly into the circulation, acting against pathogens and other threats in the bloodstream [94,95,96,97,98,99,100,101,102]. As other cells and organs have been described as sources of complement system proteins as well, additional functions have been ascribed to this system. Here, we highlight the importance of intracellular and secreted complement activation in disease conditions, with an emphasis on its consequences for the development of kidney fibrosis [92,103,104].

Classical pathway activation is mediated by the binding of the C1 complex (C1q/C1r/C1s) to immunoglobulins that are bound to target cell membranes. The lectin pathway is activated through the recognition of specific carbohydrates located on the pathogen surface and, finally, the alternative pathway, which is constantly activated at low levels and allows for the generation of C3b. All three pathways converge to a common, terminal pathway, leading to the assembly of the membrane attack complex (MAC), a membrane pore responsible for killing pathogens and/or target cells [95,96,97,99,100]. Cleavage of the central complement protein C3 generates the small fragments C3a and C3b, which become part of the C5 convertases that cleave C5 into C5a and C5b. Complement C6 and C7 bound to C5b form a complex that inserts into the plasma membrane, which acts as a scaffold for the assembly of C8 and multiple C9 molecules, culminating in MAC formation [101].

In a model of kidney fibrosis induced by UUO C57Bl/6, mice knockout for C1qA and C3 genes have an obvious increase in the production and deposition of ECM proteins, augmented production of α-SMA, reduction of E-cadherin, and increased production and secretion of TGF-β1 from ex vivo pericytes, compared to sham mice. In mice depleted of C1q (C1q^−^/^−^), an increase in C1s/C1r proteases and diminished levels of C3 during kidney fibrosis were observed. The depletion of C1r in another mouse model causes an evident reduction in C3 production and activation. These findings indicate the relationship between C1r/C1s and C3 production and activation in the fibrotic kidney [92,104]. 

During complement activation, fragments derived from C3 and C5—known as C3a and C5a or anaphylatoxins—are generated. They bind to their respective receptors, leading to a pro-inflammatory response, such as NLRP3 activation by C5a/C5aR2 and the production of IL-1β and IFN-γ by TCD4^+^ cells [105]. C3aR is expressed in renal epithelial cells, and its activation is related to the production of the pro-inflammatory cytokines IL-1β, IL-6, IL-8, and TNF-α by immune cells; these cytokines may exert deleterious functions during renal damage, leading to kidney fibrosis [106]. 

There is growing evidence regarding the participation of C3a and C5a in the occurrence and progression of kidney fibrosis. HK-2 cells express markers of EMT and overproduce ECM molecules when cultured for 72 hours with C3a or C5a. Cells display strong staining for α-SMA and lose positive staining for E-cadherin in immunocytochemistry after incubation with both complement anaphylatoxins [31]. 

In a study of kidney transplantation from mice lacking the CR1-related gene/protein y (Crry; a membrane-associated complement regulatory protein that limits C3 activation) and C3 (C3^−/−^) to C3aR-, C5aR-, or C3aR/C5aR-deficient mice, the C3aR^−/−^ mice show better renal function, less inflammatory cell infiltration, tubular dilatation, and atrophy, compared to C5aR^−/−^, C3aR/C5aR^−/−^, and WT transplanted mice. Interestingly, C5aR- and C3aR/C5aR-deficient mice show signs of renal failure, indicating that C3aR but not C5aR is involved in the poor outcome during renal complement activation. These findings highlight the involvement of the C3a/C3aR pathway in kidney inflammation, leading to subsequent kidney fibrosis [107]. 

### 2.4. TGF-β1 versus HGF Balance 

**Figure 3:**
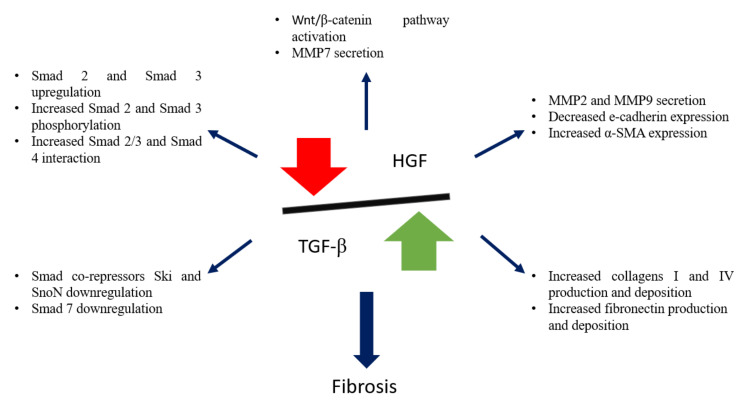
Schematic representation of TGF-β1 and HGF balance, and its importance in kidney fibrosis.

There is a close relationship and a fine balance between TGF-β1 and the multi-functional cytokine HGF (Figure 3), and the crosstalk between these pathways will be further discussed.

HGF is a pleiotropic growth factor that promotes diverse cell responses, such as mitosis, motility, and morphogenesis, as well as wound healing, tissue regeneration, tumorigenesis, and invasion. The primary structure of the protein, first studied as a hepatocyte mitogenic protein in cell cultures, was deduced more than 30 years ago [108,109]. The pathway has only one ligand—the HGF molecule—and only one receptor—the MET. 

Although some bacteria are able to subvert and bind the receptor to enter the cell, there are no other endogenous ligands for MET, except for HGF itself [74,110]. HGF is composed of an alpha and a beta chain and needs to be cleaved to its active form by serine proteases, mostly by the HGF-activator. HGF is produced and secreted by mesenchymal cells [37,69,70,74,111]. MET is a tyrosine kinase receptor (TKR), which becomes phosphorylated in multiple tyrosine residues after binding HGF, and then recruits proteins from different pathways (e.g., PI3K and Akt), which exert their roles in the activated cells (i.e., suppression of cell death by expression of anti-apoptotic Bcl-xl, inhibition of Fas-FasL binding, and inhibition of caspase3-mediated apoptosis) [112,113,114,115]. Other roles of the HGF–MET pathways are dictated by the activation of RAS/ERK pathways, which regulate cell proliferation and motility [111]. 

A fine-tuned relationship between HGF and TGF-β1 has been reported both in vivo and in vitro, with respect to different cell types and animal models. EMT and fibrosis are consequences of an imbalance of this relationship, leading to the production and secretion of hallmark proteins [39,40,111,116,117,118,119]. As EMT is induced by the exogenous administration of TGF-β1 in HK-2 cells, Wei et al. [116] evaluated the action of HGF from mesenchymal stem cells (MSC) in counteracting the effects of TGF-β1. After stimulation with the growth factor and co-culture of HK-2 cells and MSC, signs of EMT reduce, and E-cadherin and α-SMA return to basal levels. To prove that HGF is responsible for the blockade of EMT, MSC were transfected with siRNA for HGF, and there was no blockage, showing that HGF was necessary for the results [116]. 

In the renal fibrotic process, this balance is deregulated and leans towards TGF-β1, which is one of the causes of kidney fibrosis. In a mouse model of congenital nephrosis, kidney local expression and secretion of TGF-β1 is augmented while HGF levels are diminished, showing that a fine-tuned regulation of these two growth factors is required to maintain homeostasis and avoid kidney fibrosis [37]. HGF counteracts TGF-β1 by inducing the expression of SnoN, one of the co-repressors of the TGF-β1 pathway. 

In a study using a cellular model, EMT and ECM production are induced through the stimulation of cells with purified TGF-β1. Cells were treated with TGF-β1 in the presence or absence of HGF, and whole-cell lysates were analyzed by western blot. When treated with both TGF-β1 and HGF, there is an increase in SnoN production, but no reduction in Smad2 phosphorylation. By co-immunoprecipitation, it was shown that SnoN and p-Smad2 are associated, indicating that SnoN physically binds to p-Smad2 and impedes the activation of target genes [120]. 

### 2.5. Renin-Angiotensin-Aldosterone System and Its Relation with Fibrosis 

Despite its well-known role in maintaining blood volume, blood pressure, and Na^+^ levels within normal values, the renin-angiotensin-aldosterone system (RAAS) has been implicated in organ fibrosis, mainly by its capacity to activate the TGF-β1 signaling pathway [121,122,123]. 

Angiotensin II (Ang II) directly binds to cellular receptors and activates important pathways (e.g., NF-κB and ERK). One of the described targets for Ang II is TLR4, known for its affinity for bacterial LPS. Renal dysfunction is characterized by augmented serum creatinine, albuminuria, and blood urea nitrogen, and kidney fibrosis hallmarks include increased collagens I/IV deposition and elevated levels of TGF-β1 and MMP9. Knocking down MD2—a TLR4 accessory protein that mediates binding of the ligand to its receptor—or blocking it with a small inhibitor molecule leads to a decrease in renal dysfunction and kidney fibrosis seen in wild type mice after the subcutaneous injection of Ang II. It also affects cytokine and chemokine production mediated by NF-κB and ERK pathways. Of note, all the effects observed upon Ang II administration are MD2 dependent, thus, indicating that Ang II signals using the TLR4/MD2 complex [123]. 

The binding of Ang II to its type-1 receptor (AT1R) is apparently involved in the occurrence of EMT in HK-2 cells induced by high glucose medium, and mediated by the effector proteins mTOR and p70S6K. Silencing the receptor rescues the cells from EMT, characterized by overexpression of E-cadherin, diminished levels of α-SMA, and reduced expression of EMT core transcription factors [124]. Conversely, activation of the pathway by AT2R has the opposite effects and protects cells from TGF-β1 activation by inhibiting TGFβRII, and consequently, preventing EMT [125]

Ang II is capable of inducing secretion, and activating the latent form of TGF-β1, thus, having a pro-fibrotic role [126]. Ang II activates Smad2 and Smad3, which will enter the nucleus and display the same effects already described upon activation by TFG-β1 binding to its receptor, such as an enhanced production of the TGF-β1 molecule, leading to positive feedback [122]. 

Although this pathway is well-described and associated to renal fibrosis in different pathological contexts [123,124,125,126,127], the involvement of RAAS in kidney fibrosis due to chronic infection by pathogenic *Leptospira*, as deep as it was possible for us to search, has not been addressed, but may be a possible subject of investigation regarding the sequelae from leptospiral infection. 

### 2.6. EMT, a Hallmark of Kidney Fibrosis 

As research involving EMT has grown in the past 20 years, it has become almost mandatory to elaborate guidelines to help researchers in formulating projects and to develop concepts that could aid in eliminating controversies regarding terms associated to EMT. In June 2020, an extensive committee, on behalf of The EMT International Association, published the *Guidelines and definitions for research on epithelial–mesenchymal transition* [128]. 

The epithelial to mesenchymal transition is a commonly described phenomenon that takes place during three major events: embryogenesis, cancer, and fibrosis. It is described as a process that occurs after specific stimuli in epithelial cells, leading to the acquisition of mesenchymal phenotype markers and a loss of epithelial markers. Although it may seem to be a static and end-point event, there is a spectrum regarding epithelial to mesenchymal phenotype markers, and, in fibrosis, cells mostly undergo an incomplete EMT, retaining characteristics that resemble both epithelial and mesenchymal phenotypes [128]. 

Although still controversial, there is evidence that the partial EMT of tubular cells participates in renal fibrosis indirectly, as EMT leads to an increased production of cytokines and chemokines, such as TGF-β1, that stimulate interstitial fibroblasts to produce more ECM proteins and, as such, attract more immune cells and enhance local inflammation [129,130,131,132].

In vitro and in vivo studies have also described the occurrence of EMT or partial EMT in tubular epithelial cells. The expression of mesenchymal markers (e.g., α-SMA), loss of epithelial markers (e.g., E-cadherin), increased production of ECM proteins (e.g., fibronectin and collagens I, III, and IV), and over- and down-regulation of specific genes (e.g., *Zeb1* and *Zeb2*, *Snai1,* and *Snai2*) are indicative of this process, pointing to the active participation of epithelial cells in the occurrence and progression of kidney fibrosis [23,116,132,133,134]. 

Although necessary during renal embryogenesis, *Snai1* and *Snai2* are completely inactive during adulthood and are only reactivated during fibrosis. Tubular epithelial cells from mice subjected to UUO show an increased expression of *Snai1*, while there is an evident and important increase in interstitial fibrosis concomitant with a reduction in the expression of epithelial markers, such as cadherin 1. In *Snai1^fl/fl^* mice, fibrosis, and mesenchymal markers are clearly less evident, and epithelial markers are still perceived in tubular epithelial cells, demonstrating that Snai1 is necessary for partial EMT in those cells. 

The contribution of tubular epithelial cells to the generation of myofibroblasts (ECM-producing cells) in kidney fibrosis is a controversial issue among EMT researchers. Using a mouse model expressing the Tomato fluorescent protein and UUO to induce fibrosis, it became clear that tubular cells undergo partial EMT, but do not delaminate and detach from the basement membrane to invade the interstitium, as less than 1% of interstitial cells were tdTomato^+^ 7 days after obstruction [130].

Mice lacking *Twist1* or *Snail1* genes have 48.2% and 64.3% lesser tubular epithelial cells, respectively, that underwent EMT after UUO compared to wild type mice. In addition, those mice displayed less collagen deposition and better renal function. No signs of tubular epithelial cell detachment are seen both in wild type and knockout mice, indicating that these cells underwent partial EMT and that they are needed to induce α-SMA^+^ myofibroblasts. EMT in kidney fibrosis is associated with a lower expression of important solute transporters, such as aquaporin 1 and Na^+^/K^+^-ATPase, and suppressing the expression of core EMT-transcription factors may explain the better renal function in *Twist1* or *Snail1* KO mice [135]. 

EMT is under strict control in adult humans and animals [128,130,131,136]. One of the regulators that helps keep EMT under control are micro-RNAs (miRNAs), especially the family of miRNAs known as miR-200, which downregulate the expression of *Zeb1* and *Zeb2*. Both transcription factors are overexpressed during fibrosis and EMT, and downregulate the expression of E-cadherin [137,138,139]. In a rat tubular epithelial cell line model, EMT is induced by TGF-β1, while miR-200a is downregulated in a Smad2-dependent way. When miR-200a is overexpressed, cells are protected from TGF-β1-induced EMT, indicating that Smad2 is necessary and controls the expression of miR-200a [138]. With that in mind, the use of miR-200 family precursors as a possibility for kidney fibrosis treatment and/or retardation of fibrosis development has been tested, with some good results [140]. 

## 3. Acute Leptospirosis and the Development of Kidney Fibrosis 

During infection, pathogenic *Leptospira* disseminate through the bloodstream and reach target organs, especially the kidneys, liver, and lungs [141]. The kidneys are then colonized by the bacteria and elicit an immunological response, initially based on the recruitment of neutrophils and further mediated by macrophages. Meanwhile, leptospires are still found in the blood in the first three days after infection, where neutrophils try to eliminate them by NETosis and other mechanisms. 

Neutrophil-depleted mice have a higher leptospiral burden in the kidneys 15 days post-infection, which demonstrates that neutrophils help to eliminate *Leptospira* from the blood within the first few days [142,143]. Although both cell types are phagocytic and play important roles in the innate immune response to invading pathogens, *Leptospira* has the ability to subvert and scape phagocytosis, invade renal convoluted tubules, and persist in this niche [144,145,146,147,148]. 

The transition of acute kidney injury (AKI) caused by leptospiral infection to chronic infection and, consequently, kidney fibrosis is still not completely understood, and many research gaps remain open. This is also applied to the transition from AKI to CKD of non-infectious cause [149]. Continuous stimuli, the activation of inflammatory pathways, and the participation of innate immune cells in the development of chronic leptospirosis and kidney fibrosis are the main underlying causes that have been considered so far [25,142,143,149,150,151].

The sustained stimulus caused by the presence of *Leptospira* in the convoluted tubules is responsible for the overactivation of inflammatory pathways, triggering pro-fibrotic signals. One month after infection, cellular infiltration in the kidneys is characterized by CD3-positive T-cells and CD11b-positive macrophages/monocytes, but no more neutrophils are observed [152]. In a rat model of gentamicin-induced acute kidney injury, the most prevalent cell infiltrate in kidneys were pro-inflammatory M1 macrophages within the first day after injury; whereas, at day 30 after injury, M2 macrophages accounted for 45% of the total cell infiltrate [149]. 

The total healing of the kidney, with no signs of tubular necrosis or glomerular sclerosis, can also be observed. Activation of the NF-κB and NLRP3/IL-1β pathways are implicated in the recrudescence of signs in rat kidneys 180 days after injury, with a low-grade inflammation occurring together with activation of Angiotensin II, as well as collagen and fibronectin deposition in the interstitium. These findings indicate that sustained inflammatory stimulus may lead to kidney fibrosis after acute injury [149].

As described above, the complement is another important system activated during leptospirosis. Fluid-phase or membrane-associated negative complement regulatory proteins avoid overactivation of this system and, consequently, tissue damage [98,101,105]. One of the membrane-associated complement regulators is the decay-accelerating factor 1 (Daf1), responsible for inhibiting the assembly and accelerating the disassembly of C3 and C5 convertases. In a mouse model of leptospirosis infection, Daf1^−/−^ mice have higher bacterial loads, greater susceptibility to infection, acute renal lesions, and more evident kidney fibrosis 90 days post-infection, with more tubulointerstitial collagen deposition than wild-type littermates. These findings point to a role of Daf1 in controlling the bacterial burden and inflammation during the acute phase, thus, helping to reduce chronic lesions and fibrosis [150].

Cytotoxicity mediated by nitric oxide (NO) is one of the mechanisms used by macrophages to control leptospiral infection. Use of the TLR2/NOD2 agonist CL429 increases NO production by mice peritoneal and bone marrow-derived macrophages when exposed to *L. interrogans* serovars Manilae str. L495, Copenhageni str. Fiocruz L1-130, and Icterohaemorraghiae str. Verdun [151]. The NO increase is correlated with a lower number of live *Leptospira* in cell culture and, as such, is associated with bacterial killing. Inducible nitric oxide synthase (iNOS) expression—and, consequently, NO production—is also associated with kidney fibrosis as the disease transitions from an acute to chronic state in C57BL/6J mice [152].

Of note, the initial lesion and the type of cellular infiltrate play roles in the disease progression and, thus, contribute to the evolution to chronic and fibrotic *Leptospira*-related kidney disease [142,150,152]. The roles of macrophages and galectin-3 in the survival rate and clinical course of the disease, acute interstitial nephritis, and development of chronic infection and kidney fibrosis in C57BL/6 mice infected by *L. interrogans* sorovar Copenhageni str. L1-130 have been investigated [142]. Although galectin-3 plays a crucial role in controlling bacterial burden during the acute phase, fibrosis and chronic disease is only correlated with the initial bacterial burden, being directly related to the development and extent of kidney fibrosis, leading to the activation and enrichment of fibrosis-related pathway genes [25,142,152].

In brief, acute leptospirosis is characterized mainly by inflammatory and immune responses mediated by cells.

## 4. Kidney Fibrosis and Chronic Leptospirosis

Chronic leptospiral infection leads to kidney fibrosis, but the underlying mechanisms and pathways involved are still poorly elucidated. In the first part of this review, the potential pathways related to renal fibrosis were described. In this section, we will address the current knowledge on the findings related to renal fibrosis caused by *Leptospira* infection. 

Hamsters and guinea pigs are considered good animal models for acute and severe leptospirosis, as both animals die within the first 5–10 days after *Leptospira* inoculation [141,153,154]. On the other hand, rats and mice are suitable models to study carrier status and chronic disease. Although mice do not present signs of the disease, and their lesions are considered mild to moderate, good models of chronic leptospirosis in those animals have been developed, while some underlying mechanisms involved in the persistence of *Leptospira* and fibrosis induction have begun to be elucidated [142,150,152,155].

Using wild-type and different knockout mouse lineages to understand which pathways may be activated during chronic leptospiral infection, Fanton d´Andon et al. [152] demonstrated that renal fibrosis in chronic mouse infection can be partially attributed to nitric oxide production but in a TLR- and NLR-independent manner. Furthermore, acute inflammation and T-cell infiltration do not contribute directly to the extent of renal fibrosis. 

Chronic leptospiral infection has been associated to fibrosis in many different models of mouse infection [142,150,152,156]. Both wild-type and decay-accelerating factor 1-deficient mice (DAFKO) developed fibrosis at 90 days post-infection, where collagen deposition is observed away from lymphocyte infiltration. In contrast to what has been previously described [152], the authors found a relationship between inflammation and interstitial fibrosis in both wild-type and DAFKO mice, but with more evident collagen deposition in the interstitium of DAFKO mice, compared to the wild-type [150]. 

*Leptospira* outer membrane proteins induce the accumulation of ECM proteins in renal epithelial cells through activation of the TGF-β1/Smad3 pathway, thus, contributing to the evolution of fibrosis associated to chronic infection [132]. Therefore, TGF-β1 presents a profibrotic action and is involved in the mechanisms of renal fibrosis during chronic leptospirosis. According to Tian et al. [157], outer membrane proteins from *L. santarosai* serovar Shermani enhance the secretion of collagen types I and IV by HK-2 cells, and the process is mediated by the TGF-β1 pathway. 

Bone marrow derived-macrophages can transdifferentiate into myofibroblasts—cells that are involved in the secretion of α-SMA. This transition is coordinated by the TGF-β1/Smad3 pathway, in a process known as the macrophage–myofibroblast transition (MMT). In chimeric mice that had their bone marrow depleted by radiation and reconstituted by exogenous GFP-expressing C57BL/6 bone marrow, GFP-positive myofibroblasts were observed in the kidneys after UUO. 

To ascertain that the MMT occurs, orchestrated by TGF-β1/Smad3, C57BL/6 mice were irradiated and reconstituted with bone marrow from GFP^+^Smad3^+/+^ and GFP^+^Smad3^−/−^. Mice lacking Smad3 did not present bone marrow-derived myofibroblasts after UUO surgery, thus, corroborating the involvement of TGF-β1/Smad3 in the MMT within the kidney [158]. 

To understand which pathways are involved in chronic infection and kidney fibrosis caused by *L. interrogans* serovar Copenhageni str. L1-130 and the role of leptospiral infection in progression of CKD, Chou et al. [25] performed a mouse kidney transcriptomic analysis and detected increased gene expression of TGF-β1, Wnt, and integrin-β—crucial players in important fibrosis-related pathways. In addition, when mice are submitted to a nephrotoxic diet with 0.1 or 0.2% of adenine, those pathways are further enriched. 

Comparing orthologous genes from mice and humans with leptospiral infection plus nephrotoxic stimulus and CKDu, respectively, there is an overlap of enriched genes. These findings provide support for the hypothesis that *Leptospira* infection is associated with CKD progression and may be an underlying cause of CKDu. Furthermore, as both Wnt and TGF-β1 pathways are enhanced in this model, it is suggested that they contribute to the progression of kidney fibrosis [25].

Further evidence of a fibrotic process triggered by infection with leptospires comes from recent in vitro and in vivo findings from our group. HK-2 cells infected with *L. interrogans* serovar Manilae str. L495 were shown to produce greater amounts of fibronectin and collagen type IV, compared to non-infected cells. Morphological alterations, such as spindle shape, loss of cell–cell contact, cell grouping, and abundant ECM production, were also evident in infected HK-2 cells. Cellular and molecular mechanisms underlying this EMT process are under investigation. 

**Figure 4:**
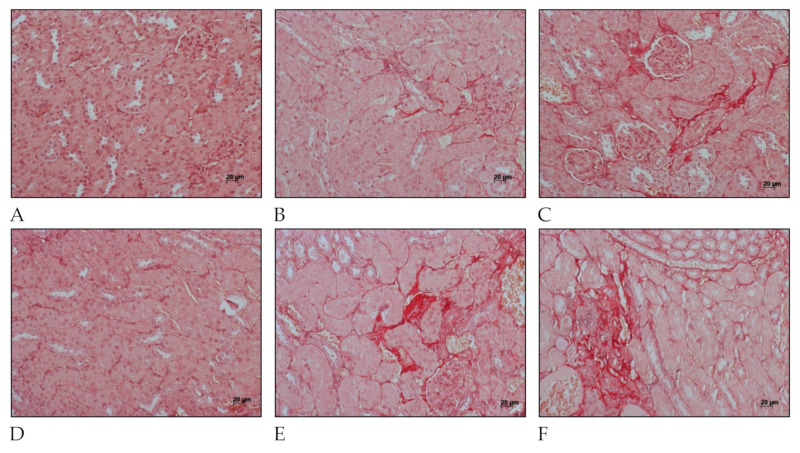
Histology micrography from C57BL/6 mice kidneys infected by *Leptospira interrogans* sorovar Manilae str, L495. Collagen deposition is evidenced by red staining in peritubular and perivascular areas and the interstitium. (**A**,**D**) Negative control injected with 200 µL of PBS, 15 days post-infection (d.p.i.) and 30 d.p.i, respectively; (**B**,**C**) Kidneys of mice injected with 1 × 10^6^ *Leptospira* and 2 × 10^8^ *Leptospira*, respectively, at 15 d.p.i.; (**E**,**F**) Kidneys of mice injected with 1 × 10^6^ *Leptospira* and 2 × 10^8^ *Leptospira*, respectively, at 30 d.p.i.; Picrosirius Red staining, lateral bar = 20 µm.

**Figure 5:**
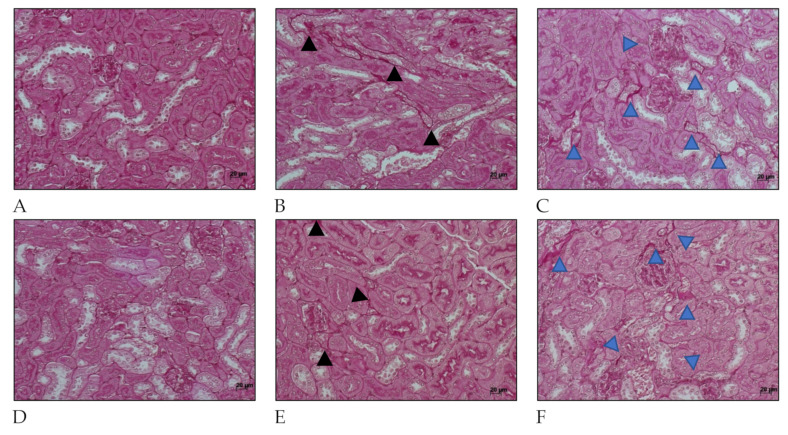
Histology micrography from C57BL/6 mice kidneys infected by *Leptospira interrogans* sorovar Manilae str. L495. Collagen deposition is evidenced by red staining in peritubular areas and the interstitium. (**A**,**D**) Negative controls injected with 200 µL of PBS 15 days post infection (d.p.i.) and 30 d.p.i, respectively; (**B**,**C**) Kidneys of mice injected with 1 × 10^6^ *Leptospira* and 2 × 10^8^ *Leptospira*, respectively, at 15 d.p.i.; (**E**,**F**) Kidney from mice injected with 1 × 10^6^ *Leptospira* and 2 × 10^8^ *Leptospira*, respectively, at 30 d.p.i.; Periodic acid–Schiff staining; black and blue arrow heads: collagen deposition; lateral bar = 20 µm.

Fibrosis-related histopathological alterations in the kidneys of C57BL/6 mice after *L. interrogans* infection are shown in Figure 4 and Figure 5. Collagen deposition, evidenced by Picrosirius Red staining in peritubular areas and the interstitium, were already detected at day 15 post-infection and became more pronounced and widely distributed in the renal tissue at day 30 post-infection in kidneys of mice infected with *L. interrogans* serovar Manilae str. L495 (Figure 4). 

Collagen deposition was also seen in Periodic acid–Schiff (Figure 5) at 15 days after infection in kidney considering mice infected with serovar Manilae str. L495. After 30 days of infection, mice that were infected with a high amount (2 × 10^8^) of *Leptospira* showed a few more areas of interstitial, and peritubular fibrosis (indicated by blue arrow heads) than those infected with less (1 × 10^6^) *Leptospira* (indicated by black arrow heads). These findings show the importance of this serovar in causing acute disease as well as chronic and profibrotic infection.

## 5. Kidney Fibrosis and the CKDu Outbreak—The Relationship with Leptospirosis

As CKD has become one of the most prevalent kidney diseases, due to systemic hypertension or diabetes, it is worth highlighting two major points: (*i*) impaired kidney function and fibrosis are present in almost every case of CKD, and (*ii*) the outcome of CKD is renal failure, requiring kidney transplantation. Highlighting those aspects is of utmost importance, as CKD patients demand constant care, such as dialysis, hospital care, and transplantation, thus, elevating healthcare expenditures [18,19].

According to a recent systematic analysis, 1.2 million people died from CKD and 697.5 million CKD cases were recorded globally in 2017, with a global prevalence of 9.1% [159]. The burden is higher in countries located in Oceania, Sub-Saharan Africa, and Latin America, following a pattern already described in other studies [16,19]. Of note, those regions accounted for the highest rate of leptospirosis cases worldwide [2,7]. Without any further analysis, it is noticeable that regions with a higher incidence of CKD and leptospirosis cases overlap. In the past few years, evidence that humans may remain chronic carriers of *Leptospira* has given rise to a discussion about how it could be related to the endemic increase in CKD and CKDu [160,161,162].

Another recent systematic review of observational studies has disclosed a correlation between leptospirosis and CKD. Poor renal function, characterized by a low estimated glomerular filtration rate (eGFR), is more prevalent in anti-*Leptospira* positive individuals than in negative ones, and those with higher titers of antibodies against *Leptospira* have poorer renal function (lower eGFR) [163].

In a study considering a Peruvian community, 189 out of 314 asymptomatic participants had high titers of anti-*Leptospira* antibodies and 13 were leptospiral 16S rRNA-positive by PCR from urine samples. Despite being asymptomatic, *Leptospira* molecular detection in those individuals evidence that the chronic carrier state is a reality. Although the likelihood of renal damage and kidney fibrosis was not assessed in this work, the presence of *Leptospira* in the kidneys may be a risk factor for the development of CKD [160]. In a study of canine leptospirosis, a relevant and close correlation between CKD and leptospirosis has been reported; furthermore, those dogs with CKD and leptospirosis are more frequently associated with *Leptospira* shedding [164].

Mesoamerican nephropathy, a type of CKDu, has brought to light evidence that chronic leptospirosis could be a cause of CKD. This condition is highly prevalent in male sugarcane workers that are in close contact with flooded areas at least during a period of the year. It is proposed that leptospirosis may be one of the causes (but not the only) of Mesoamerican nephropathy, and that exposure to other potentially nephrotoxic conditions or substances may influence the occurrence of the disease [165]. Recently, a study conducted in a mouse model of chronic leptospirosis, followed by toxic exposure to alimentary adenine, demonstrated that renal lesions and fibrosis are more severe and prevalent in mice primarily infected by *Leptospira*, showing that chronic leptospirosis may be a risk factor for the development of CKD [25].

## 6. Gaps and Perspectives

CKD/CKDu are important health problems that directly impact the quality of life and health systems all over the world. Despite recent advances in nephrology and conscientization campaigns, the searches for medical assistance and treatment are still insufficient. Assuming that leptospirosis may play a role in the development of CKD/CKDu, and considering the overlap of both conditions in Asia, Central and South America, and Sub-Saharan Africa, studies into the associated pathophysiology, cellular communication pathways, and treatment strategies to avoid or control the fibrotic process are needed. 

As discussed throughout the text, kidney fibrosis and, consequently, CKD, as a sequela of *Leptospira* infection, is a well-documented manifestation of the disease; however, studies on the mechanisms associated with the pathophysiology of leptospiral renal fibrosis are still scarce and deserve to be deepened. Deciphering the cellular communication pathways in *Leptospira*-induced kidney fibrosis may shed light on the specific actors involved in this process.

## Data Availability

The data presented in the article are available in the article.
