# Correction: Prado, L.G.; Barbosa, A.S. Understanding the Renal Fibrotic Process in Leptospirosis. *Int. J. Mol. Sci.* 2021, *22*, 10779

**DOI:** 10.3390/ijms23169233

**Published:** 2022-08-17

**Authors:** Luan Gavião Prado, Angela Silva Barbosa

**Affiliations:** 1 Laboratório de Bacteriologia, Instituto Butantan, Avenida Vital Brasil, 1500, São Paulo 05503-900, Brazil; 2 Departamento de Microbiologia, Instituto de Ciências Biomédicas, Universidade de São Paulo, Avenida Lineu Prestes 1374, São Paulo 05508-000, Brazil

The authors wish to make the following corrections to this paper [1]:

The activation of fibrosis-related pathways, more specifically of the TGF-β1 pathway, has been reported in in vitro assays with cell lines. However, different results were found using in vivo models [2,3,4]. Therefore, some statements throughout the text are equivocal as they do not refer to in vivo data.

The following sentences or fragments of the text should be deleted:

Page 10: “(e.g., iNOS, TGF-β1, Wnt, and integrin)”

Page 10: “but it triggers the initial activation of the above-mentioned pathways, such as TGF-β1 and Wnt/β-catenin. Their sustained and imbalanced activation will then promote major effects on the occurrence of EMT and kidney fibrosis during infection [25,132,149].”

Page 10: “Thus far, the involvement of the TGF-β1 and Wnt/β-catenin pathways have been associated with fibrosis resulting from chronic leptospiral infection; however, the picture is certainly more complex, and we must keep in mind that other pathways may contribute to fibrosis caused by this spirochete [153–155].”

Page 14: “As the evidence that pathways described herein (i.e., TGF-β1 and Wnt/β-catenin) are directly associated to fibrosis and the progression of CKD in humans and in mice previously infected by *L. interrogans* becomes clearer, it is questionable whether chronic and asymptomatic leptospiral infection is related to CKDu, as well as whether it contributes to the progression of CKD to end-stage kidney disease.”

Page 14: “The supposed role of TGF-β1 in fibrosis induced by leptospiral components, such as LipL32 or outer membrane proteins in human kidney epithelial cells, has been evaluated [134], but no studies have been conducted using live *Leptospira* in cellular models. Furthermore, animal models of infection have focused mainly on gross and histopathological alterations, and additional studies addressing which pathways are altered during chronic leptospiral infection will help in preventing and treating important leptospirosis sequelae.”

Reference corrections:

- Reference [153] on page 10 referring to the paragraph “Cytotoxicity mediated by nitric oxide (NO) is one of the mechanisms used by macrophages to control leptospiral infection. Use of the TLR2/NOD2 agonist CL429 increases NO production by mice peritoneal and bone marrow-derived macrophages when exposed to *L. interrogans* serovars Manilae str. L495, Copenhageni str. Fiocruz L1-130, and Icterohaemorraghiae str. Verdun” is incorrect. The correct citation is reference [151].
- Alterations to the reference citations in the main text (highlighted in bold):

Page 11: “Hamsters and guinea pigs are considered good animal models for acute and severe leptospirosis, as both animals die within the first 5–10 days after *Leptospira* inoculation [141,**153**,**154**].”

Page 11: “Although mice do not present signs of the disease, and their lesions are considered mild to moderate, good models of chronic leptospirosis in those animals have been developed, while some underlying mechanisms involved in the persistence of *Leptospira* and fibrosis induction have begun to be elucidated [142,150,152,**155**].”

Page 11: “Chronic leptospiral infection has been associated to fibrosis in many different models of mouse infection [142,150,152,**156**].”

Page 11: “According to Tian et al. [157], outer membrane proteins from *L. santarosai* serovar Shermani enhance the secretion of collagen types I and IV by HK-2 cells, and the process is mediated by the TGF-β1 pathway.”

Page 11: “Mice lacking Smad3 did not present bone marrow-derived myofibroblasts after UUO surgery, thus, corroborating the involvement of TGF-β1/Smad3 in the MMT within the kidney [**158**].”

Page 13: “According to a recent systematic analysis, 1.2 million people died from CKD and 697.5 million CKD cases were recorded globally in 2017, with a global prevalence of 9.1% [**159**].”

Page 13: “In the past few years, evidence that humans may remain chronic carriers of *Leptospira* has given rise to a discussion about how it could be related to the endemic increase in CKD and CKDu [**160–162**].”

Page 13: “Poor renal function, characterized by a low estimated glomerular filtration rate (eGFR), is more prevalent in anti-*Leptospira* positive individuals than in negative ones, and those with higher titers of antibodies against *Leptospira* have poorer renal function (lower eGFR) [**163**].”

Page 13–14: “Although the likelihood of renal damage and kidney fibrosis was not assessed in this work, the presence of *Leptospira* in the kidneys may be a risk factor for the development of CKD [**160**].”

Page 14: “In a study of canine leptospirosis, a relevant and close correlation between CKD and leptospirosis has been reported; furthermore, those dogs with CKD and leptospirosis are more frequently associated with *Leptospira* shedding [**164**].”

Page 14: “It is proposed that leptospirosis may be one of the causes (but not the only) of Mesoamerican nephropathy, and that exposure to other potentially nephrotoxic conditions or substances may influence the occurrence of the disease [**165**].”

- Changes in the Reference list (from Ref. [153] on):

153. Richer, L.; Potula, H.H.; Melo, R.; Vieira, A.; Gomes-Solecki, M. Mouse Model for Sublethal Leptospira Interrogans Infection. *Infect. Immun.* **2015**, *83*, 4693–4700.

154. Gomes-Solecki, M.; Santecchia, I.;Werts, C. Animal Models of Leptospirosis: Of Mice and Hamsters. *Front. Immunol.* **2017**, *8*, 58.

155. Werts, C. Murine Models for Leptospirosis Kidney Disease. *Transl. Res. Biomed.* **2019**, *7*, 65–75.

156. Chassin, C.; Picardeau, M.; Goujon, J.-M.; Bourhy, P.; Quellard, N.; Darche, S.; Badell, E.; d’Andon, M.F.; Winter, N.; Lacroix-Lamandé, S.; et al. TLR4- and TLR2-Mediated B Cell Responses Control the Clearance of the Bacterial Pathogen, Leptospira Interrogans. *J. Immunol.* **2009**, *183*, 2669–2677.

157. Tian, Y.-C.; Hung, C.-C.; Li, Y.-J.; Chen, Y.-C.; Chang, M.-Y.; Yen, T.-H.; Hsu, H.-H.; Wu, M.-S.; Phillips, A.; Yang, C.-W. Leptospira Santorosai Serovar Shermani Detergent Extract Induces an Increase in Fibronectin Production through a Toll-Like Receptor 2-Mediated Pathway. *Infect. Immun.* **2011**, *79*, 9.

158. Wang, S.; Meng, X.; Ng, Y.; Ma, F.Y.; Zhou, S.; Zhang, Y.; Yang, C.; Huang, X.; Xiao, J.;Wang, Y.; et al. TGF-β/Smad3 Signalling Regulates the Transition of Bone Marrow-Derived Macrophages into Myofibroblasts during Tissue Fibrosis. *Oncotarget* **2015**, *7*, 8809.

159. Bikbov, B.; Purcell, C.A.; Levey, A.S.; Smith, M.; Abdoli, A.; Abebe, M.; Adebayo, O.M.; Afarideh, M.; Agarwal, S.K.; Agudelo-Botero, M.; et al. Global, Regional, and National Burden of Chronic Kidney Disease, 1990–2017: A Systematic Analysis for the Global Burden of Disease Study 2017. *Lancet* **2020**, *395*, 709–733.

160. Ganoza, C.A.; Matthias, M.A.; Saito, M.; Cespedes, M.; Gotuzzo, E.; Vinetz, J.M. Asymptomatic Renal Colonization of Humans in the Peruvian Amazon by Leptospira. *PLoS Negl. Trop. Dis.* **2010**, *4*, e612.

161. Herath, N.J.; Kularatne, S.A.M.; Weerakoon, K.G.A.D.; Wazil, A.; Subasinghe, N.; Ratnatunga, N.V.I. Long Term Outcome of Acute Kidney Injury Due to Leptospirosis? A Longitudinal Study in Sri Lanka. *BMC Res. Notes* **2014**, *7*, 1–4.

162. Monahan, A.M.; Callanan, J.J.; Nally, J.E. Review Paper: Host-Pathogen Interactions in the Kidney during Chronic Leptospirosis. *Vet. Pathol.* **2009**, *46*, 792–799.

163. Carrillo-Larco, R.M.; Altez-Fernandez, C.; Acevedo-Rodriguez, J.G.; Ortiz-Acha, K.; Ugarte-Gil, C. Leptospirosis as a Risk Factor for Chronic Kidney Disease: A Systematic Review of Observational Studies. *PLoS Negl. Trop. Dis.* **2019**, *13*, e0007458.

164. Sant’Anna, R.; Vieira, A.S.; Oliveira, J.; Lilenbaum, W. Asymptomatic Leptospiral Infection Is Associated with Canine Chronic Kidney Disease. *Comp. Immunol. Microbiol. Infect. Dis.* **2019**, *62*, 64–67.

165. Correa-Rotter, R.; Wesseling, C.; Johnson, R.J. CKD of Unknown Origin in Central America: The Case for a Mesoamerican Nephropathy. *Am. J. Kidney Dis.* **2014**, *63*, 506–520.

The authors apologize for any inconvenience caused and state that the scientific conclusions are unaffected. The original publication has also been updated.
